# Implementation of an Opioid and Benzodiazepine Deprescribing Program for Older Adults in an Outpatient Setting

**DOI:** 10.1093/geroni/igaa057.3334

**Published:** 2020-12-16

**Authors:** Lori Armistead, Jan Busby-Whitehead, Stefanie Ferreri, Cristine Henage, Tamera Hughes, Jack McBride, Ellen Roberts, Ben Urick

**Affiliations:** 1 ^1.^ University of North Carolina Eshelman School of Pharmacy, Chapel Hill, North Carolina, United States; 2 University of North Carolina School of Medicine, Chapel Hill, North Carolina, United States

## Abstract

The United States spends $50 billion each year on 2.8 million injuries and 800,000 hospitalizations older adults (age 65 years and older) incur as the result of falls. Chronic use of central nervous system (CNS)-active medications, such as opioid and/or benzodiazepine (BZD) medications, increases the risk of falls and falls-related injuries in this older adult population. This Centers for Disease Control and Prevention (CDC)-funded randomized control trial uses electronic health record (EHR) data from primary care outpatient clinics to identify older adult patients at risk for falls due to chronic opioid or BZD use. The primary program aim is to test the efficacy of a targeted consultant pharmacist service to reduce the dose burden of these medications in the targeted population. Impact of this intervention on the risk of falls in this population will also be assessed. Licensed clinical pharmacists will review at-risk patients’ medical records weekly and make recommendations through the EHR to primary care providers for opioid or BZD dose adjustments, alternate medications, and/or adjunctive therapies to support deprescribing for approximately 1265 patients in the first two cohorts of intervention clinics. One thousand three hundred eighty four patients in the control clinics will receive usual care. Outcome measures will include reduction or discontinuation of opioids and BZDs and falls risk reduction as measured by the Stop Elderly Accidents, Death and Injuries (STEADI) Questionnaire. Primary care provider adoption of pharmacists’ recommendations and satisfaction with the consult service will also be reported.

